# Supplementary material for: Peripheral opioid receptor antagonism alleviates fentanyl-induced cardiorespiratory depression and is devoid of aversive behavior
Source: eLife. 2025 Apr 1;13:RP104469. doi: 10.7554/eLife.104469 (PMC11961120; doi:10.7554/eLife.104469)
Supplement: Supplementary file 1. — (A) Baseline cardiorespiratory values in rats prior to receiving 20 µg/kg fentanyl. Values are mean ± SE. Data are from rats used in Figures 1, 3 and 4 and are separated by sex. There was no significant difference in any of the measured parameters between male and female rats. Oxygen saturation: one-way ANOVA, F(5,27) = 0.6787, p = 0.6424; heart rate: one-way ANOVA, F(5,27) = 1.078, p = 0.3944; respiratory rate: one-way ANOVA, F(5,27) = 1.527, p = 0.2147. (B) Baseline cardiorespiratory values in male and female rats prior to receiving 50 µg/kg fentanyl. Values are mean ± SE. Data are from rats used in Figure 1, Figure 3—figure supplement 1, and Figure 4—figure supplement 1 and are separated by sex. Female rats in Figure 1 had a significantly higher heart rate at baseline compared male rats in Figure 1. Oxygen saturation: one-way ANOVA, F(5,25) = 1.161, p = 0.3558; heart rate: one-way ANOVA, F(5,25) = 3.774, p = 0.0110, Tukey’s post hoc test †p < 0.05 Figure 1 males versus Figure 1 females; respiratory rate: one-way ANOVA, F(5,25) = 1.728, p = 0.1649. (C) Nadir and recovery values in male and female rats that received 20 µg/kg fentanyl. Values are mean ± SE. Data are from rats used in Figures 1, 3, and 4 and are separated by sex. For nadir data: oxygen saturation: one-way ANOVA, F(5,27) = 1.229, p = 0.3230; heart rate: one-way ANOVA, F(5,27) = 1.844, p = 0.1379; respiratory rate: one-way ANOVA, F(5,27) = 2.669, p = 0.0438. Despite the significant interaction for respiratory rate, no post hoc differences were detected. Sex differences in recovery times were also evaluated. oxygen saturation: one-way ANOVA, F(5,27) = 0.7021, p = 0.6267; heart rate: one-way ANOVA, F(5,27) = 0.4536, p = 0.8069; respiratory rate: one-way ANOVA, F(5,27) = 1.100, p = 0.3832. (D) Nadir and recovery values in male and female rats that received 50 µg/kg fentanyl. Values are mean ± SE. Data are from rats used in Figure 1, Figure 3—figure supplement 1, and Figure 4—figure supplement 1 and are se [file elife-104469-supp1.docx]

| **Supplementary File 1A. Baseline cardiorespiratory values in male and female rats prior to receiving 20 µg/kg fentanyl** | Figure 1: Dose Response | | Figure 3: Pretreatment | | Figure 4: Reversal | |
| --- | --- | --- | --- | --- | --- | --- |
|  | Males | Females | Males | Females | Males | Females |
| Oxygen Saturation Baseline (%) | 94 ± 0.5 | 95 ± 0.4 | 96 ± 0.4 | 96 ± 0.4 | 95 ± 0.6 | 95 ± 0.3 |
| Heart Rate Baseline (beats per minute) | 445 ± 16 | 426 ± 13 | 462 ± 12 | 439 ± 17 | 436 ± 10 | 416 ± 13 |
| Respiratory Rate Baseline (breaths per minute) | 109 ± 6 | 95 ± 3 | 108 ± 5 | 114 ± 3 | 103 ± 4 | 102 ± 4 |
| **Supplementary File 1B. Baseline cardiorespiratory values in male and female rats prior to receiving 50 µg/kg fentanyl** | Figure 1: Dose Response | | Figure 3 – Figure  Supplement 1 | | Figure 4 – Figure  Supplement 1 | |
|  | Males | Females | Males | Females | Males | Females |
| Oxygen Saturation Baseline (%) | 94 ± 1 | 95 ± 1 | 94 ± 1 | 96 ± 1 | 94 ± 1 | 95.2 ± 1.1 |
| Heart Rate Baseline (beats per minute) | 408 ± 10 | 485 ± 13* | 450 ± 21 | 434 ± 14 | 421 ± 17 | 451 ± 33 |
| Respiratory Rate Baseline (breaths per minute) | 95 ± 2 | 110 ± 4 | 101 ± 5 | 98 ± 4 | 100 ± 1 | 105 ± 8 |

| **Supplementary File 1C. Nadir and recovery values in male and female rats that received 20 µg/kg fentanyl** | Figure 1: Dose Response | | Figure 3: Pretreatment | | Figure 4: Reversal | |
| --- | --- | --- | --- | --- | --- | --- |
|  | Males | Females | Males | Females | Males | Females |
| Oxygen Saturation Nadir (%) | 50 ± 4 | 50 ± 4 | 42 ± 6 | 38 ± 1 | 53 ± 6 | 45 ± 2 |
| Heart Rate Nadir (beats per minute) | 182 ± 26 | 144 ± 10 | 253 ± 32 | 177 ± 55 | 226 ± 45 | 146 ± 20 |
| Respiratory Rate Nadir (breaths per minute) | 59 ± 3 | 49 ± 4 | 67 ± 4 | 45 ± 12 | 66 ± 5 | 60 ± 4 |
| Oxygen Saturation Recovery (minutes) | 11 ± 1 | 10 ± 1 | 12 ± 2 | 14 ± 2 | 14 ± 3 | 11 ± 1 |
| Heart Rate Recovery (minutes) | 15 ± 13 | 16 ± 1 | 15 ± 3 | 13 ± 5 | 15 ± 3 | 18 ± 1 |
| Respiratory Rate Recovery (minutes) | 12 ± 2 | 14 ± 1 | 10 ± 1 | 17 ± 4 | 11 ± 1 | 15 ± 3 |

| **Supplementary File 1D. Nadir and recovery values in male and female rats that received 50 µg/kg fentanyl** | Figure 1: Dose Response | | Figure 3 – Figure Supplement 1 | | | Figure 4 – Figure  Supplement 1 | |
| --- | --- | --- | --- | --- | --- | --- | --- |
|  | Males | Females | Males | | Females | Males | Females |
| Oxygen Saturation Nadir (%) | 30 ± 3 | 37 ± 2 | | 41 ± 4 | 35 ± 6 | 40 ± 5 | 38 ± 4 |
| Heart Rate Nadir (beats per minute) | 181 ± 16 | 145 ± 20 | | 159 ± 17 | 179 ± 37 | 135 ± 17 | 188 ± 33 |
| Respiratory Rate Nadir (breaths per minute) | 48 ± 6 | 41 ± 5 | | 56 ± 10 | 55 ± 9 | 50 ± 8 | 61 ± 6 |
| Oxygen Saturation Recovery (minutes) | 31± 6 | 28 ± 7 | | 30 ± 5 | 22 ± 6 | 29 ± 5 | 28 ± 6 |
| Heart Rate Recovery (minutes) | 23 ± 5 | 48 ± 1 | | 43 ± 9 | 33 ± 5 | 35 ± 7 | 41 ± 1 |
| Respiratory Rate Recovery (minutes) | 16 ± 3 | 43 ± 1^† #^ | | 26 ± 7 | 18 ± 4 | 13 ± 4 | 38 ± 3 |
